# Injury Prevalence of the Lower Limbs in Handball Players: A Systematic Review

**DOI:** 10.3390/ijerph19010332

**Published:** 2021-12-29

**Authors:** Iván Martín-Guzón, Alejandro Muñoz, Jorge Lorenzo-Calvo, Diego Muriarte, Moisés Marquina, Alfonso de la Rubia

**Affiliations:** 1 Facultad de Ciencias de la Actividad Física y del Deporte (INEF—Departamento de Deportes), Universidad Politécnica de Madrid, C/Martín Fierro 7, 28040 Madrid, Spain; ivanmartinguzon@gmail.com (I.M.-G.); jorge.lorenzo@upm.es (J.L.-C.); diego.muriarte@upm.es (D.M.); moises.mnieto@upm.es (M.M.); 2 Exercise and Sport Sciences, Faculty of Health Sciences, Universidad Francisco de Vitoria, 28223 Pozuelo de Alarcón, Spain

**Keywords:** handball, injury, knee, ankle, body area, tissue, pathology, epidemiology

## Abstract

Lower limb injuries are frequent in handball and a serious hindrance to athletic performance. The aim of this systematic review was to synthesize the available research on the prevalence of lower limb injuries in handball players according to sex and competitive level. According to the Preferred Reporting Items for Systematic Reviews and Meta-Analysis, 19 studies were selected after a systematic search and selection process of three digital databases: Scopus, PubMed, and Web of Science. Furthermore, a study quality analysis using an ‘Extension for Sports Injury and Illness Surveillance of the Strengthening the Reporting of Observational Studies in Epidemiology (STROBE-SIIS)’ was carried out. The sample consisted of 7110 male and female handball players registering 4483 injuries in their lower limbs. The results showed a high incidence of knee injuries (30.23%) and ankle injuries (24.80%), especially in the ligaments, such as the talofibular and the anterior cruciate ligaments. Considering sex and competitive level, knee injuries accounted for 47.02% of injuries among women, while among men, ankle injuries were most prevalent (34.22%) in international competitions. Additionally, the most common cause of injuries was trauma (85.61%). The findings highlighted that the prevalence of lower limb injuries varies greatly according to the characteristics of the sample and injury. Therefore, the study underlines the importance that sports practitioners (physical trainers, readapters, and physiotherapists) adapt training protocols to reduce injury incidence in the most affected body areas or tissues.

## 1. Introduction

Handball is a high-intensity, high-impact sport that can cause a great deal of aerobic and anaerobic stress in players [1], although the frequent impacts and body contact do not generally lead to total breaks [2]. With the great variability of repetitive movement (accelerations, braking, sprints, changes of direction, jumps, throws, thrusts, and pulls) performed at maximum speed and with a high degree of instability and uncertainty [3], handball can be considered a sport with a high risk of injury compared to other team and individual sports [4].

The increasing speed of play, the increasing number of matches per season, and the growing physical and physiological demands on the musculoskeletal system in the game have increased the injury risk for handball players [5]. Scientific evidence has shown a higher prevalence of injury in the lower limbs than in the upper limbs, head, and neck [6,7]. The unilateral nature of play actions, instability, and contact for aerial situations, is the most important cause of acute injuries or trauma to the lower limbs (e.g., ligament sprains and bone fractures), while the high speed of muscle contraction required by certain movements and displacements cause the majority of injuries due to overuse/overload (e.g., muscle breaks and tears) [8]. The first type of injury is caused by the sudden onset of the pathology or pain, which in handball are usually caused by landing and throwing actions [9]. On the other hand, the term ‘overuse injury’ is commonly applied to gradual-onset injuries that lack a definable sudden, precipitating event [10], which co-occur with sprinting or other high-intensity displacement actions (e.g., change of direction). Another determinant factor in handball injuries is fatigue due to the accumulation of playing time during matches [11]. In fact, Langevoort et al. [6] confirmed that the highest number of injuries suffered in six international handball championships occurred between minute 11 and 20 of the first half and minute 41 and 50 of the second half, that is, in moments when play becomes most intense and the players more tired.

As for the location of lower limb injuries, knee and ankle injuries were most common [12]. A study of 109 handball players from the top two leagues in Iceland confirmed that the most injured joints in the lower limb were the knee (26%) and ankle (19%) [13]. Although greater protection for the player, the result of changes in regulations by the International Handball Federation (IHF) [14] has resulted in this type of injury occurring to a greater extent in non-contact situations [7]. These situations, produced in situations without any direct or indirect contact from other external sources [15], often lead to severe cases with long recovery periods, especially in the knee joint and the thigh and leg muscle groups [16]. Furthermore, more frequent non-contact situations in handball (i.e., a feint or landing action on a single) [17] are usually associated with intrinsic risk factors, such as previous injuries and athletic or regenerative deficits [12].

With regard to injury patterns, there are several determining factors of the number and type of injuries in handball. Firstly, it has been found that the incidence of injury differs according to sex [18]. Specifically, Olsen et al. [19] confirmed that the risk of suffering an *anterior* cruciate *ligament* (ACL) rupture in the knee was higher among women than men. Other variables were age or competition category [20]. The latest scientific evidence in this regard, based on heterogeneous results, found no differences between senior players and those competing in formative categories [20,21,22]. Finally, the sports context in which the injury occurs was also analyzed. The risk of injury during competition is 3 to 10 times higher than in a training or preparatory session [7]. It also appears that the severity of the injury and the length of the recovery period are greater if the injury takes place during a match [23].

To our knowledge, no systematic review of lower limb injuries in handball players has been conducted. Considering the number of injuries in sport, and specifically in handball, due to many factors [24], and its importance to athletic performance, the aim of this systematic review was to synthesize the available scientific evidence regarding the prevalence/incidence of injury in the lower limb (body area, tissue type, pathology type, and injury mechanism) in handball players according to sex and competitive level.

## 2. Materials and Methods

The stages of the review procedure and subsequent analysis of the original articles stayed within the guidelines set out in the Preferred Reporting Items for Systematic Reviews and Meta-Analysis (PRISMA) checklist and the Population, Interventions, Comparisons, Outcomes, and Study Design (PICOS) question model for the definition of inclusion criteria [25,26].

### 2.1. Study Selection and Eligibility Criteria

The systematic review included original studies published in peer-review journals with an impact factor included in the Journal Citation Report of the Web of Science (JCR—WoS) or Scimago Journal and Country Rank (SJR of Scopus).

The inclusion criteria, according to the ‘PICOS’ model (Population, Interventions, Comparisons, Outcomes, and Study Design) [25], were: (1) Population: high-level professional handball players participating in national or international official competitions (1st or 2nd level of competition) and professional players involved in talent identification and development systems—TID systems (3rd level of competition) [27]; (2) Intervention: studies with information on injuries caused during official competition in the lower limbs of handball players; (3) Comparison: relationship between the injury characteristics and player characteristics of sex and competitive level; (4) Results: prevalence and type of injury in the lower limbs of handball players; (5) Study design: analytical-descriptive approach of a non-experimental nature to determine the incidence of injuries to the lower limb in handball players.

The exclusion criteria were: (1) counting the number of injuries in individual sports or other collective sports (football, basketball, rugby, etc.), also excluding beach handball; (2) evaluation of injuries to the upper limbs, trunk, neck, and/or head; (3) exclusive analysis of the mechanics or kinematics of the lesion; (4) injury prevention study or testing; (5) using a sample including amateur or semi-professional players and in formative categories; (6) absence of specific information about the player characteristics; (7) type of format of the scientific document (lecture, letter to the editor, book chapter, case study). In addition, systematic reviews were not included but used only as a source of information.

### 2.2. Data Search Process and Systematic Review Protocol

The selection process of scientific studies was conducted by searching three electronic databases: PubMed, Web of Science, and Scopus. The terms, in English, were grouped into three search strings: (1) ‘injury*’ or ‘sprain*’ or ‘dislocation*’ or ‘fracture*’ or ‘rupture*’ or ‘concussion*’ or ‘tear*’ or ‘strain*’ or ‘tendinosis’ or ‘arthritis’ or ‘bursitis’; and (2) ‘handball’ or ‘associative sport*’ or ‘collaborative sport*’ or ‘handball player*’ or ‘professional handball player*’ or ‘elite handball player*’; and (3) ‘lower body*’ or ‘lower limb*’ or ‘lower extremity*’ or ‘anterior cruciate ligament’ or ‘leg’ or ‘groin’ or ‘hip*’ or ‘pelvis’ or ‘buttock*’ or ‘thigh’ or ‘knee*’ or ‘lower leg’ or ‘ankle*’ or ‘foot’ or ‘feet’ or ‘toe*’.

**Figure 1:**
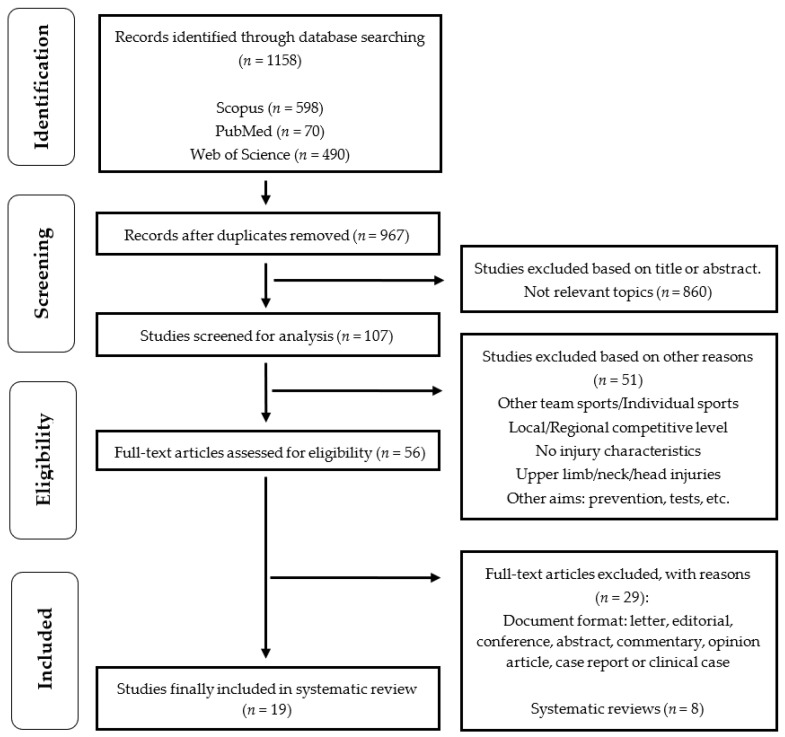
Flow chart for screening and selection according to Preferred Reporting Items for Systematic Reviews and Meta-Analysis (PRISMA).

According to the criteria for conducting systematic reviews, ‘Preferred Reporting Items for Systematic Reviews and Meta-Analysis’—PRISMA [26], the study was carried out during May and September 2021 and was composed of four stages (Figure 1): (1) Identification: the first (I.M.G.) and sixth author (A.d.l.R.) found 1,158 studies in the three databases analyzed (Scopus, PubMed, and Web of Science); (2) Screening: duplicate studies (*n* = 191) and those considered non-relevant based on an initial reading of the title, abstract and keywords (*n* = 860) were eliminated by the second (A.M.) and third author (J.L.C.). Furthermore, these same authors rejected 51 scientific papers for ‘other reasons’ (see Figure 1). At the end of this phase, there were 56 remaining studies. (3) Eligibility: the fourth (D.M.) and fifth author (M.M.) eliminated those studies that did not have an original article published in a peer-review scientific journal (*n* = 29) and also systematic reviews or meta-analyses (*n* = 8); (4) Inclusion: a total of 19 studies were considered suitable for analysing and synthesizing their research.

### 2.3. Data Extraction and Management

The information extracted from the studies ultimately included in this systematic review was classified according to three research variables to establish the following categories and subcategories: (A) Sample characteristics: (A1) number of players; (A2) sex (male and female); and (A3) competitive level (international or 1st level, national or 2nd level and TID systems or 3rd level); (B) Injury characteristics: (B1) body area: ‘hip’ (groin, pelvis and buttocks), ‘thigh’ (anterior thigh, posterior thigh and lateral thigh), ‘knee’ (anterior cruciate ligament—ACL, posterior cruciate ligament—PCL, medial collateral ligament—MCL, lateral meniscus, medial meniscus, knee cartilage and patellar tendon), ‘lower leg’ (tibia, fibula, anterior tibial, fibula, twin muscles, and Achilles tendon), ‘ankle’ (talus, talofibular ligaments and peroneal tendon) and ‘foot’ (sole, cuneiforms and metatarsals); (B2) type of tissue (muscle, bone, ligament or tendon); (B3) pathology type; and (B4) injury mechanism (trauma or overuse). The injury characteristics were classified according to the International Olympic Committee’s Consensus Declaration on Injuries and Diseases [15]. Evidence of the damaged structure was not included in all studies due to the absence of exact specifications. The information on the pathology type is partial given that in these studies, the data refer to a set of injuries (including head/neck/trunk and upper limb) or the set of sports analyzed, not specifically distinguishing the injuries of the lower limb in handball. Data were presented using the absolute frequencies (*n*) extracted from each study.

### 2.4. Study Quality Assessment

To analyse the quality of the studies, an ‘Extension for Sports Injury and Illness Surveillance of the Strengthening the Reporting of Observational Studies in Epidemiology (STROBE-SIIS)’ checklist [15] was used. This checklist was composed of 23 items clustered into six categories belonging to the different study sections: ‘Title—Abstract’ (item 1), ‘Introduction’ (items 2–3), ‘Methods’ (items 4–12), ‘Results’ (items 13–17), ‘Discussion’ (items 18–21), and ‘Funding’ (items 22–23). A score of ‘0′ was assigned to incomplete items, and ‘1′ to items that were described accurately. The overall rating was obtained from the summation of the item values based on the following levels: ‘very low quality’ (0–4 points), ‘low quality’ (5–8 points), ‘medium quality’ (9–12 points), ‘high quality’ (13–16 points), and ‘very high quality’ (17–23 points). The quality assessment of the studies was carried out by two independent reviewers (I.M.G. and A.d.l.R.). The other four reviewers (A.M., J.L.C., D.M., and M.M.) resolved disagreements in the rating, and inter-rater reliability was calculated.

## 3. Results

The scientific evidence drawn from this systematic review is provided in Table 1. It shows, in chronological and alphabetical order, the 19 studies providing data on the sample characteristics (‘*n*’, sex, age, and competitive level) and the injury characteristics (‘*n*’, body area, tissue type, pathology type, and injury mechanism).

### 3.1. Sample Characteristics

The sample consisted of 7110 handball players, of whom 76.83% (*n* = 5465) were men and 23.14% (*n* = 1645) were women. The mean age of the entire sample was 23.2 ± 4.9. With regard to the competitive level, four studies were identified that were carried out in international sports contexts (*n* = 931), nine in national competitions (*n* = 2862), one related to TID systems (*n* = 42), and five in which two competitive levels were present simultaneously, that is, a study with international and national level players (*n* = 1899) and four studies with a sample of national level players belonging to TID systems (*n* = 1376).

### 3.2. Injury Characteristics

A total of 4483 injuries or pathologies in the lower limb were recorded. In relation to injuries characteristics (Table 2), the most injured body areas in handball players were the knee (30.23%) and ankle (24.80%). Here, the most common injuries were ligamentous, both in the knee (*n* = 164) and in the ankle (*n* = 160). It should be noted that in a high percentage of cases, while the location was specified, the tissue type suffered was not specified (85.66%).

**Figure 2:**
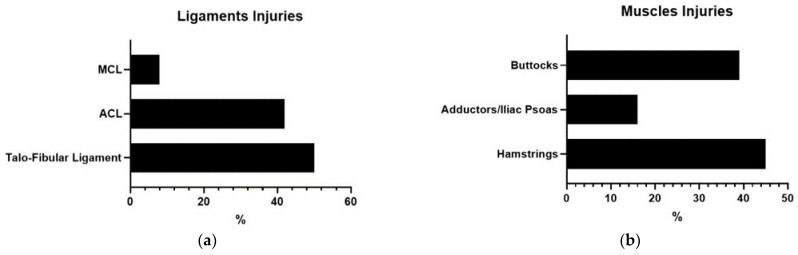

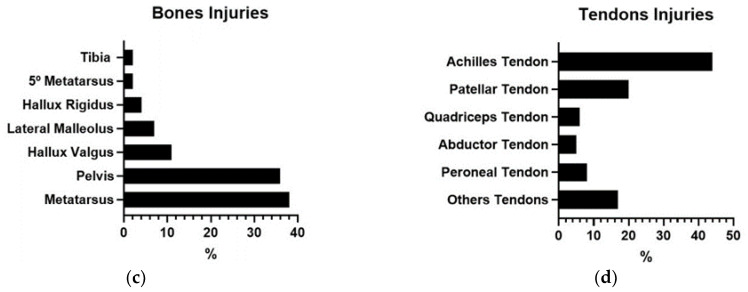
(**a**–**d**) Type of pathology in the lower limb based on the tissue injured.

The most commonly damaged tissues, depending on the body area injured, were (Figure 2a–d): (1) Ligaments. The ligaments with the highest incidence were the talofibular ligaments of the ankle (50.00%) and the ACL (42.00%); (2) Muscles. The most common injuries occurred in the upper posterior part of the lower limb, that is, the hamstrings (45.00%) and the buttocks (39%); (3) Bones. The most affected bones were, in this order, the metatarsal bones of the foot (38.00%) and the pelvis (36.00%); (4) Tendons. The highest number of pathologies were in the Achilles tendon (44.00%).

### 3.3. Injury Prevalence

Table 3 shows the injury incidence among the handball players, according to body area. In terms of sex, 82.73% of injuries were recorded in men’s competitions, among which there was a higher number of knee (23.63%) and ankle (21.42%) injuries, while in the female competition, only 9.40% of injuries were registered, with the knee joint being the most affected (4.42%). As for the competitive level, the highest number of injuries occurred in national competitions (82.52%), followed by international competitions (7.27%) and TID systems formative categories (2.34%). In international competitions (level 1), there was a high incidence of ankle injuries (*n* = 114), while in levels 2 and 3 (national competitions and TID systems), there were high incidences of both knees (26.20%) and ankle (21.04%). Analysing both factors together (sex and competitive level), it was observed that knee (*n* = 939; 20.95%) and ankle (*n* = 827; 18.45%) injuries were most common in men’s national competitions while knee (*n* = 195; 4.35%) injuries were the most prevalent in women’s national competitions. The studies showed that injuries in female international competitions accounted for only 0.56% and within TID systems for 0.07%. It was recorded 351 injuries as ‘not codable’ because they did not provide information on the sex of the players, accounting for 7.87% of the total.

**Figure 3:**
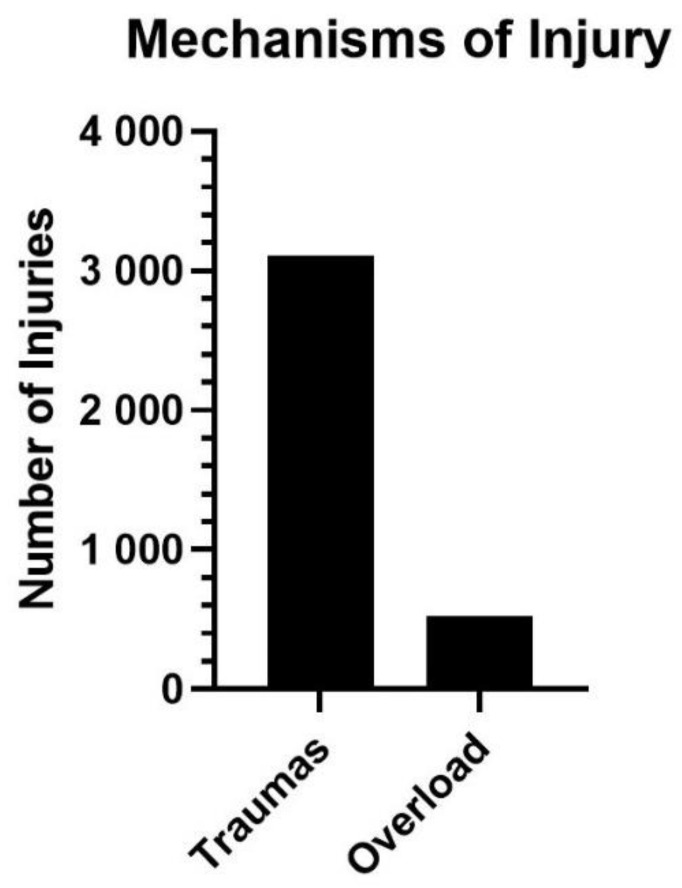
Production mechanism of lower limb injuries among handball players.

Associated with the mechanism of injury production (Figure 3), there were 3105 injuries caused by trauma or sudden impacts (85.61%), while only 522 (14.39%) were caused by overload or overuse of the structure or tissue. A high percentage of trauma injuries (68.72%) were identified in men’s national competitions. In women, the cause of the injury was only reported in 60 of the cases registered and analysed.

### 3.4. Study Selection and Assessment (Quality Analysis)

The quality analysis (‘Extension for Sports Injury and Illness Surveillance of the Strengthening the Reporting of Observational Studies in Epidemiology (STROBE-SIIS)’ checklist) yielded the following results (Table 4): (a) the quality scores ranged from 12 to 22; (b) the average score was 16.47 points; (c) of the 19 included studies, 1 (5.26%) was considered ‘medium quality’ (9–12 points); 5 (26.32%) were categorized as ‘high quality’ (13–16 points); and 13 (68.42%) were considered ‘very high quality’ (17–23 points); (d) the highest scores (19 points) were located in items no. 1, no. 3, no. 5, no. 10, no. 18 and no. 20. By contrast, the most commonly absent or incomplete items were no. 4 (5 points), no. 17, and no. 22 (6 points).

## 4. Discussion

The present study represents the most comprehensive and exhaustive systematic review of the prevalence of lower limb injuries in handball players. The main strengths of this research lie in the qualities of external validity and generalisability of the results from the analysis of a large sample of handball players (*n* = 7110) and injuries (*n* = 4483), as well as a strict evaluation of the published data. Furthermore, from a methodological perspective, this systematic review presents a robust design based on the search process carried out in four databases and limited to unambiguously inclusion and exclusion criteria. The results showed: (i) a high incidence of injuries to the knee (30.23%) and ankle (24.80%), especially in ligamentous structures such as the talofibular ligaments and the ACL; (ii) knee injuries accounted for almost half of the pathologies recorded in women (47.02%), higher than in men (28.56%); (iii) in international men’s competitions, the number of ankle injuries (*n* = 103) was slightly higher than knee injuries (*n* = 83); (iv) most injuries are caused by trauma (85.61%).

The parts of the body most prone to injury are those that bear the most load during play. This is the case of lower limbs in handball, where knees and ankles suffer the highest rate of injuries. These results coincide with the results of previous research [4,38]. Poorly executed specific actions, such as feints or landings after a jump [17], and the predominantly unilateral nature of these actions [39] are two of the most important risk factors. When there is not an optimal range of movement for executing technical physical actions, the player will compensate with passive or support structures, resulting in postural imbalances and increasing the risk of injury [31]. Specifically, these passive structures corresponded to the knee and ankle joints, the ACL and the APL, respectively.

One of the most common injuries to the lower limbs was partial or total rupture of the ACL. However, in the present study and coinciding with other studies [19,40,41], the incidence was almost three times higher among women than men. Similarly, the incidence of LCA increased the higher the level of competition [42]. One differentiating factor appears to be the activation capacity of the hamstring muscles [43]. Female handball players, in landing (e.g., jumping/suspension throwing), even while showing higher electromyographic levels in the quadriceps than men [44], are not able to recruit a necessary number of fibers in the hamstring musculature to stabilize the knee joint thus avoid excessive rotational movements [45]. Furthermore, as Schmitz et al. [46] demonstrated, the ability to absorb impacts with only one leg is lower in women, resulting in higher levels of muscle tension, ultimately affecting the ACL. Finally, a greater ‘Q angle’ in women, due to a wider pelvis and shorter femur, produces greater tension forces on the knee ligaments and, especially, on the ACL [47,48].

Another structure with a high injury incidence is the hamstrings. This muscle group is prone to injury due to rapid accelerations, pivots, and/or feints by handball players [30]. Hamstrings may shorten due to daily physical activity and suffer a loss of viscoelasticity, possibly resulting in postural changes that modify the original action of these muscles and cause pathologies [49]. Moreover, high levels of fatigue in this muscle group appear to be related to an increased risk of ACL injury [50]. Thus, there is a logical prevalence of hamstring injuries among handball players. On the other hand, the Achilles tendon, due to jumping and physical overload [35], and the metatarsals, due to stress fractures [51], were also identified as structures prone to injury.

With regard to the injury mechanism, the results of the present study correspond to those of previous research into handball [6,52]. Considering this is a high-contact sport [53], it is not surprising that most injuries to the lower limbs are caused by trauma (85.61%) due to either direct or indirect contact. Frequent actions such as changes of direction in reduced spaces, feints, 1 × 1 actions, and landing after jumps tend to increase the injury prevalence by trauma [9]. The joint most affected by this type of injury mechanism is the ankle, generally due to actions where a teammate/opponent is stepped on after a defensive blocking action (breaks) or in the take-off prior to a throw (sprains) [54]. Conversely, injuries to the hip, thigh, or lower leg would be most associated with overuse, being shielded from physical contact with other players [51]. Nevertheless, findings were mixed with regard to the knee [55]. The evidence suggests that injuries are often due to trauma (e.g., rupture of the ACL) [19], while clinical cases show that pathologies are caused by overuse (e.g., chronic inflammation of the patellar tendon [56].

With regard to differentiation by competitive level, a higher incidence of ankle injuries was found in international men’s competitions. Two explanatory factors are the greater intensity and speed of the game, leading to higher levels of fatigue, and the direct relationship between a player’s age and the risk of suffering ankle injuries [20]. However, as Bere et al. [7] pointed out in their study on the Men’s Handball World Championship in 2015, most ankle injuries were sprains without severity. In relation to injuries in players involved in TID systems, it would be appropriate to focus on an increasingly higher incidence, even higher than in senior categories [57]. This fact seems to be due, to a large extent, to the lower maturational development of young players that would lead to a lower predisposition of the body structures to withstand the impacts/contacts that occur in a sport such as handball [20,58]. Other factors considered as key were a reduced ability to avoid injury-prone actions due to a lower skill level, maladjustment to the competition conditional requirements, and a lack of individualized prevention/recovery strategies and processes in youth competitive categories [11].

### 4.1. Limitations

To our knowledge, this is the first systematic review to analyse specifically the injuries of the lower limb in handball. However, some limitations are present such as: (i) a reduced number of participants in the female samples and international contexts or TID systems associated with lower limb injuries; (ii) lack of accuracy in injury identification and collection processes (e.g., video recording or past injury questionnaire); (iii) lack of definition or lack of specificity with regard to data relating to the injury characteristics (e.g., pathology type or production mechanism); (iv) absence of a register of other parameters related to lower limb injuries (e.g., recovery time, recurrence, etc.); (v) no calculate of the injury index given the players’ exposure time (in game, training, or total).

### 4.2. Practical Applications

This study constitutes a starting point for further research into the prevalence of lower limb injuries among handball players. Some of the possible practical applications are: (i) focus on preventive measures by physical trainers, readapter and physiotherapists specialized in areas and structures of the lower limb with a higher prevalence of injuries; (ii) personalization of training loads according to sex and competitive level by the staff; (iii) development of warm-up protocols to gain an ideal degree of activation in order to reduce the risk of injury in body areas with a high incidence; (iv) develop different effective strategies to prevent and/or cure lower limb injuries by increasing the neuromuscular activation level and achieving dynamic balance, such as strength work in all its forms and types (concentric, eccentric and isometric) [59], CORE training in all planes of body movement (sagittal, frontal and transverse) [60], multidirectional plyometric exercise, sprinting tasks and changes of direction and jumping and landing exercises [61] or proprioceptive training [62].

### 4.3. Future Lines of Research

There is still a long way to go on this topic. Future studies should collect detailed data on medical consultations and diagnostic procedures to allow the evaluation of possible biases in estimating the effects of treatment. Novel treatments such as occlusive and neuromuscular training need to be studied in order to reduce injury time and return to play. Research on the work phases carried out by the doctor, physiotherapist, rehabilitator, and physical trainer for physical and technical maintenance during the injury is also needed.

Furthermore, future studies on lower limb injuries in other team sports with similar physical demands to establish possible common production mechanisms and studies associated with psycho-emotional problems derived from injuries are required.

Finally, the establishment of a database with specific information on the players’ profile (sex, age, playing position, laterality, etc.) would be of great help to individualize the processes of prevention, treatment, and recovery of injuries in team sports.

## 5. Conclusions

Injuries to the lower limb most often occur in the knee and ankle, commonly in ligamentous structures such as the anterior cruciate ligament and the talofibular ligaments. In women’s competitions, the incidence was higher in the structures that form the knee joint, especially the anterior cruciate ligament, while in men’s competitions, a greater number of injuries to the ankle joint were observed. As for patterns in the injury production mechanism, trauma was most often caused by actions with or without contact.

Therefore, this research could provide a valid tool for practitioners (coach, physical trainer, physiotherapist, etc.) and handball players with regard to injury typology and incidence in order to implement individualized strategies and protocols for prevention, readaptation, and reincorporation to sport according to sex and competitive level.

## Figures and Tables

**Table 1:** Summary of the sample (“*n*”, sex, age, and competitive level) and injury characteristics (“*n*”, body area, tissue type, pathology type, and injury mechanism).

Study	SAMPLE CHARACTERISTICS				INJURY CHARACTERISTICS				
	*n*	Sex	Age	Competitive Level	Sex (*n*)	Body Area	Tissue Type	Pathology Type	Injury Mechanism
Badekas et al. [28]	75 30	M W	24.0	International (level 1)	M (1)	Thigh	Muscle	Hamstring	-
					M (23)/W (5)	Lower Leg	Tendon	Achilles Tendon	
					M (3)	Lower Leg	Tendon	Tibialis Anterior	
					M (1)	Lower Leg	Bone	Tibia	
					M (16)/W (7)	Ankle	Ligament	Talofibular Ligaments	
					M (1)/W (2)	Ankle	Bone	Lateral Malleolus	
					M (8)/W (2)	Ankle	Tendon	Peroneal Tendon	
					M (8)/W (3)	Foot	Bone	Metatarsals	
					W (5)	Foot	Bone	1º Metatarsal (Hallux Valgus)	
					M (2)	Foot	Bone	1º Metatarsal (Hallux Rigidus)	
					W (1)	Foot	Bone	5º Metatarsal	
Vauhnik et al. [29]	258	W	17.7 ± 3.7	National and TID System (levels 2 and 3)	W (6)	Knee	Ligament	Anterior Cruciate Ligament	Traumatism (*n* = 6)
Moller et al. [22]	125 217	M W	20.5 18.7	National and TID System (levels 2 and 3)	37 *	Hip/Groin	Muscle	Buttocks	Traumatism (*n* = 195) Overuse (*n* = 116)
					5 *	Hip/Groin	Bone	Pelvis	
					29 *	Thigh	-	-	
					87 *	Knee	-	-	
					53 *	Lower Leg	-	-	
					6 *	Lower Leg	Tendon	Achilles Tendon	
					94 *	Ankle/Foot	-	-	
Bere et al. [7]	384	M	-	International (level 1)	M (1)	Hip/Groin	Muscle	Buttocks	Traumatism (*n* = 68) Overuse (*n* = 10)
					M (5)	Hip/Groin	Bone	Pelvis	
					M (21)	Thigh	Muscle	Hamstrings	
					M (15)	Knee	-	-	
					M (8)	Lower Leg	-	-	
					M (23)	Ankle	Ligament	Talofibular Ligaments	
					M (5)	Foot	Bone	Metatarsals	
Sonnery-Cottet et al. [30]	2	M	25.2	National (level 2)	M (1)	Thigh	Tendon	Femoral Biceps	-
					M (1)	Thigh	Tendon	Semitendinosus	
Krosshaug et al. [31]	372	W	21.4	National (level 2)	W (26)	Knee	Ligament	Anterior Cruciate Ligament	Traumatism (*n* = 26)
Steffen et al. [32]	420	W	21.4	National (level 2)	W (28)	Knee	Ligament	Anterior Cruciate Ligament	Traumatism (*n* = 28)
Giroto et al. [12]	183 156	M W	24.1 ± 5.0 22.8 ± 4.2	National (level 2)	M (8)/W (10)	Hip/Groin	Muscle	Adductors/Psoas Iliacus	Traumatism (*n* = 136) Overuse (*n* = 33)
					W (2)	Hip/Groin	Muscle	Buttocks	
					M (1)/W (2)	Hip/Groin	Bone	Pelvis	
					M (19)/W (8)	Thigh	Muscle	Hamstring	
					M (14)/W (38)	Knee	-	-	
					M (2)/W (1)	Lower Leg	Tendon	Achilles Tendon	
					M (11)/W (6)	Lower Leg	-	-	
					M (21)/W (25)	Ankle	Ligament	Talofibular Ligaments	
					M (1)	Foot	Bone	Metatarsals	
Andersson et al. [33]	55	M	-	International (level 1)	M (4)	Knee	Ligament	-	Traumatism (*n* = 10)
					M (6)	Ankle	Ligament	Talofibular Ligaments	
Luig et al. [5]	1194	M	25.3 ± 5.3	National (level 2)	M (240)	Hip/Groin	-	-	Traumatism (*n* = 2,356) Overuse (*n* = 294)
					M (444)	Thigh	-	-	
					M (706)	Knee	-	-	
					M (331)	Lower Leg	-	-	
					M (650)	Ankle	-	-	
					M (279)	Foot	-	-	
Von Rossen et al. [34]	20 22	M W	17.0	TID System (level 3)	4 *	Hip/Groin	-	-	-
					9 *	Thigh	-	-	
					11 *	Knee	-	-	
					8 *	Lower Leg	-	-	
					8 *	Ankle	-	-	
Florit et al. [35]	612	M	24.3 ± 6.8	National and TID System (levels 2 and 3)	M (6)	Thigh	Tendon	Abductor Tendon	-
					M (8)	Thigh	Tendon	Cuadriceps Tendon	
					M (2)	Thigh	Tendon	Hamstring Tendon	
					M (26)	Knee	Tendon	Patellar Tendon	
					M (14)	Knee	Tendon	Other Tendons	
					M (18)	Lower Leg	Tendon	Achilles Tendon	
					M (8)	Ankle	Tendon	-	
Mónaco et al. [20]	164	M	15.3 ± 3.0	National and TID System (levels 2 and 3)	M (35)	Thigh	-	-	-
					M (34)	Knee	-	-	
					M (52)	Ankle	-	-	
Rafnsson et al. [13]	109	M	23.4	National (level 2)	M (6)	Hip/Groin	-	-	Traumatism (*n* = 40) Overuse (*n* = 11)
					M (2)	Thigh	-	-	
					M (21)	Knee	-	-	
					M (2)	Lower Leg	-	-	
					M (10)	Ankle	-	-	
					M (10)	Foot	-	-	
Tabben et al. [36]	387	M	27.4 ± 4.4	International (level 1)	M (3)	Hip/Groin	Muscle	Buttocks	Traumatism (*n* = 37) Overuse (*n* = 16)
					M (1)	Hip/Groin	Bone	Adductors/Psoas Iliacus	
					M (1)	Hip/Groin	Bone	Pelvis	
					M (12)	Thigh	-	-	
					M (14)	Knee	-	-	
					M (3)	Lower Leg	-	-	
					M (18)	Ankle	-	-	
					M (1)	Foot	-	-	
Luig et al. [9]	1899	M	-	International and National (levels 1 and 2)	M (31)	Thigh	Muscle	-	Traumatism (*n* = 193)
					M (76)	Knee	Ligament	Anterior Cruciate Ligament	
					M (24)	Knee	Ligament	Medial Collateral Ligament	
					M (62)	Ankle	Ligament	Talofibular Ligaments	
Barič et al. [23]	81 78	M W	22.6 ± 5.1	National (level 2)	W (1)	Hip/Groin	-	-	Traumatism (*n* = 10) Overuse (*n* = 42)
					M (4)/W (10)	Knee	-	-	
					M (14)/W (18)	Ankle	-	-	
					M (3)/W (2)	Foot	-	-	
Roh et al. [18]	96 92	M W	27.1 ± 4.8	National (level 2)	M (4)/W (7)	Hip/Groin	-	-	-
					M (31)/W (32)	Thigh	-	-	
					M (66)/W (90)	Knee	-	-	
					M (24)/W (26)	Lower Leg	-	-	
					M (31)/W (43)	Ankle	-	-	
					M (15)/W (16)	Foot	-	-	
Szymski et al. [37]	79	M	26.9 ± 4.8	National (level 2)	M (9)	Hip/Groin	-	-	-
					M (35)	Thigh	-	-	
					M (41)	Knee	-	-	
					M (40)	Ankle	-	-	
					M (13)	Foot	-	-	

**Note:***n* = absolute frequency; M = men; W = women; “*” = no specified the sample sex; TID = talent identification and development; “-” = not codable. The number of handball players (*n* = 7710) did not coincide with the number of injuries (*n* = 4483) because the studies analyzed were not only composed of a sample of handball players or exclusively considered injuries to the lower limb.

**Table 2:** Distribution of lower limb injuries (*n* y %) in handball players according to body area and tissue type.

Tissue Type	Body Area												TOTAL
	Hip/Groin		Thigh		Knee		Lower Leg		Ankle		Foot		
	*n*	%	*n*	%	*n*	%	*n*	%	*n*	%	*n*	%	*n* (%)
Ligament	-	-	-	-	164	3.66	-	-	160	3.57	-	-	324 (7.23)
Muscle	92	2.05	49	1.09	-	-	-	-	-	-	-	-	141 (3.15)
Bone	15	0.33	-	-	-	-	1	0.02	3	0.07	25	0.56	44 (0.98)
Tendon	-	-	18	0.40	40	0.89	58	1.29	18	0.40	-	-	134 (2.99)
Nc	271	6.05	629	14.03	1151	25.67	472	10.53	931	20.77	386	8.61	3840 (85.66)
TOTAL *n* (%)	378	8.43	696	15.53	1355	30.23	531	11.84	1112	24.80	411	9.17	4483 (100)

**Notes:***n* = absolute frequency; % = relative frequency; ‘Nc‘ = not codable.

**Table 3:** Injury prevalence (body area) of the lower limb in handball players based on sex and competitive level.

Body Area	M			W		
	International	National	TID System	International	National	TID System
	*n* (%)	*n* (%)	*n* (%)	*n* (%)	*n* (%)	*n* (%)
Hip/Groin	11 (0.25)	268 (5.98)	-	-	22 (0.49)	-
Thigh	50 (1.10)	572 (12.76)	26 (0.57)	-	42 (0.94)	-
Knee	83 (1.85)	939 (20.95)	37 (0.83)	-	195 (4.35)	3 (0.07)
Lower Leg	38 (0.85)	379 (8.45)	9 (0.20)	5 (0.11)	33 (0.74)	-
Ankle	103 (2.30)	827 (18.45)	30 (0.67)	11 (0.25)	86 (1.92)	-
Foot	16 (0.36)	321 (7.16)	-	9 (0.20)	18 (0.40)	-

**Notes:***n* = absolute frequency; % = relative frequency; M = men; W = women; TID = talent identification and development; ‘-‘ = not codable.

**Table 4:** Study quality assessment according to the Strengthening the Reporting of Observational Studies on Injury and Illness in Sport—“STROBE-SIIS”.

Author(s)	*1	*2	*3	*4	*5	*6	*7	*8	*9	*10	*11	*12	*13	*14	*15	*16	*17	*18	*19	*20	*21	*22	*23	Score
Badekas et al. [28]	1	1	1	0	1	1	1	1	0	1	0	0	1	1	1	0	1	1	0	1	0	0	1	15
Vauhnik et al. [29]	1	1	1	0	1	1	1	1	1	1	1	1	0	0	1	1	1	1	1	1	1	0	0	18
Moller et al. [22]	1	1	1	0	1	1	1	1	1	1	0	0	0	0	0	1	0	1	0	1	1	0	1	14
Bere et al. [7]	1	1	1	1	1	0	1	1	1	1	0	1	1	1	1	1	0	1	1	1	0	0	0	17
Sonnery-Cottet et al. [30]	1	1	1	0	1	1	1	0	0	1	0	0	1	0	1	1	1	1	1	1	1	0	0	15
Krosshaug et al. [31]	1	1	1	0	1	1	1	1	1	1	1	1	1	1	1	1	1	1	1	1	1	0	1	21
Steffen et al. [32]	1	1	1	0	1	1	1	0	1	1	1	1	1	1	1	1	1	1	1	1	1	0	1	20
Giroto et al. [12]	1	1	1	0	1	1	1	1	1	1	1	1	1	0	1	1	0	1	1	1	1	0	1	19
Andersson et al. [33]	1	1	1	1	1	0	1	0	1	1	1	0	1	0	1	1	1	1	1	1	1	1	0	18
Luig et al. [5]	1	1	1	0	1	0	1	1	1	1	0	1	0	0	1	1	0	1	1	1	1	1	0	16
Von Rosen et al. [34]	1	1	1	0	1	1	1	1	1	1	1	1	1	0	0	1	0	1	1	1	1	0	0	17
Florit et al. [35]	1	1	1	1	1	1	1	1	1	1	1	0	1	1	1	1	0	1	1	1	1	0	0	19
Mónaco et al. [20]	1	1	1	1	1	1	1	1	1	1	1	1	1	1	1	1	0	1	1	1	1	1	1	22
Rafnsson et al. [13]	1	1	1	0	1	1	1	1	1	1	0	1	1	1	1	1	0	1	0	1	1	0	1	18
Tabben et al. [36]	1	1	1	0	1	0	1	1	1	1	1	0	1	1	1	1	0	1	0	1	1	1	1	18
Luig et al. [9]	1	0	1	0	1	0	1	1	1	1	0	1	1	1	1	1	0	1	1	1	1	1	1	18
BariČ et al. [23]	1	1	1	0	1	0	0	0	0	1	0	0	1	0	1	0	0	1	1	1	1	1	0	12
Roh et al. [18]	1	1	1	1	1	1	1	0	1	1	0	1	1	1	1	1	0	1	1	1	1	0	1	19
Szymski et al. [37]	1	1	1	0	1	0	0	0	1	1	0	0	1	1	1	1	0	1	1	1	1	0	1	15

**Notes:** ‘0′ = item with absence or lack of information; ‘1′ = item with complete and explicit information; in Title and Abstract, *1 (Title/Abstract) = include information on the sport, athlete population (sex, age, geographic region), level of competition, and the duration of observation. In Introduction, *2 (Background) = explain the scientific background and rationale for the investigation being reported. *3 (Objectives) = state whether study was registered. Identify the registration number and database used/state the specific purpose of the study. In Methods, *4 (Study Design) = present key elements of study design early in the paper; *5 (Settings) = describe the setting, locations, and relevant dates, including periods of recruitment, exposure, follow-up, and data collection; *6 (Participants) = define the population of athletes as well as describe how they were selected and recruited; *7 (Variables) = justify why you measured your primary and secondary outcomes of interest in the specific way chosen/describe the method for identifying the health problem outcome of interest; *8 (Data Sources) = for each variable of interest, provide sources of data and details of methods of assessment (measurement). Describe comparability of assessment methods if there is more than 1 group; *9 (Bias) = clearly report any validation or reliability assessment of the data collection tools/formally acknowledge any potential biases associated with the data collection method; *10 (Study Size) = explain how the study size was arrived at; *11 (Quantitative Variables) = explain in detail how multiple injuries/illness episodes are handled both in individual athletes and across athletes/surveillance periods/specify how injury severity was calculated; *12 (Statistical Methods) = describe all the statistical methods, including those used to control for confounding/describe any methods used to examine subgroups and interactions/explain how missing data were addressed/describe any sensitivity analyses. In Results, *13 (Participants) = report numbers of individuals at each stage of study/provide the reasons for non-participation at each stage; *14 (Descriptive Data) = include details on the level of competition being observed; *15 (Outcome Data) = in observational studies, individuals will sustain more than one health problem over the surveillance period. Take care to ensure that descriptive data represent both the number of health problems and the number of athletes affected. It is important to represent effectively both the analysis and reporting of correct units for frequency data; *16 (Main Results) = report exposure-adjusted incidence or prevalence measures with appropriate confidence intervals when presenting risk measures/report details of interest, such as the mode of onset; *17 (Other Analyses) = report injury diagnosis information, including region and tissue type in tabular form. In Discussion, *18 (Key Results) = summarize key results with reference to study objectives; *19 (Limitations) = discuss limitations in the data collection and coding procedures adopted, including in relation to any risk measures calculated; *20 (Interpretation) = provide a cautious overall interpretation of results, considering objectives, limitations, multiplicity of analyses, results from similar studies, and other relevant evidence; *21 (Generalizability) = discuss the generalizability of the athlete study population, and health problem subgroups of interest, to broader athlete groups. In Other Information, *22 (Funding) = provide the source of funding and the role of the funders for the present study and, if applicable, for the original study on which the present article is based; *23 (Ethics) = outline how individual athlete data privacy and confidentiality considerations were addressed, in line with the Declaration of Helsinki.
